# Long‐Term Sequelae of COVID‐19: A Systematic Review and Meta‐Analysis of Symptoms 3 Years Post‐SARS‐CoV‐2 Infection

**DOI:** 10.1002/jmv.70429

**Published:** 2025-06-06

**Authors:** Masoud Rahmati, Raphael Udeh, Jiseung Kang, Xenia Dolja‐Gore, Mark McEvoy, Abdolreza Kazemi, Pinar Soysal, Lee Smith, Tony Kenna, Guillaume Fond, Bastien Boussat, Duy Cao Nguyen, Huyen Do, Bach X. Tran, Nicola Veronese, Dong Keon Yon, Laurent Boyer

**Affiliations:** ^1^ CEReSS‐Health Service Research and Quality of Life Center Assistance Publique‐Hopitaux de Marseille, Aix‐Marseille University Marseille France; ^2^ Department of Physical Education and Sport Sciences, Faculty of Literature and Humanities Vali‐E‐Asr University of Rafsanjan Rafsanjan Iran; ^3^ Department of Physical Education and Sport Sciences, Faculty of Literature and Human Sciences Lorestan University Khoramabad Iran; ^4^ CRSMP, Center for Mental Health and Psychiatry Research – PACA Marseille France; ^5^ School of Life Sciences, Faculty of Science University of Technology Sydney Ultimo New South Wales Australia; ^6^ School of Health and Environmental Science Korea University College of Health Science Seoul South Korea; ^7^ School of Medicine and Public Health University of Newcastle NSW Australia; ^8^ Hunter Medical Research Institute University of Newcastle New South Wales Australia; ^9^ La Trobe Rural Health School, College of Science, Health and Engineering La Trobe University VIC Australia; ^10^ Department of Geriatric Medicine, Faculty of Medicine Bezmialem Vakif University Istanbul Turkey; ^11^ Centre for Health, Performance and Wellbeing Anglia Ruskin University Cambridge UK; ^12^ Department of Public Health, Faculty of Medicine Biruni University Istanbul Turkey; ^13^ Centre for Immunology & Infection Control Queensland University of Technology Brisbane Queensland Australia; ^14^ TIMC‐IMAG, UMR 5525 Joint Research Unit, Centre National de Recherche Scientifique, National Center for Scientific Research Université Grenoble‐Alpes Grenoble France; ^15^ Institute for Global Health Innovations Duy Tan University Da Nang Vietnam; ^16^ Research Institute for Advanced Nursing (RIAN) Dong Nai Technology University Bien Hoa Vietnam; ^17^ Department of Health Policy and Management, College of Health Science Korea University Seongbuk‐gu Korea; ^18^ Faculty of Public Health VNU University of Medicine and Pharmacy, Vietnam National University Hanoi Vietnam; ^19^ International Institute for Training and Research (INSTAR) VNU University of Medicine and Pharmacy, Vietnam National University Hanoi Vietnam; ^20^ Saint Camillus International University of Health Sciences Rome Italy; ^21^ Center for Digital Health, Medical Science Research Institute, Kyung Hee University Medical Center Kyung Hee University College of Medicine Seoul Korea; ^22^ Department of Pediatrics Kyung Hee University College of Medicine Seoul Korea

**Keywords:** COVID‐19, long COVID, meta‐analysis, SARS‐CoV‐2 infection

## Abstract

The symptoms of long COVID are well‐documented. However, the long‐term effects beyond 2 years remain poorly understood due to a lack of data. This systematic review and meta‐analysis examined the prevalence of persistent symptoms in COVID‐19 survivors 3 years following initial SARS‐CoV‐2 infection. PubMed, MEDLINE (Ovid), CENTRAL, Web of Science, Scopus, and Embase were searched from inception of the databases up to July 20, 2024, by two independent researchers for articles reporting on the prevalence of persistent symptoms 3 years' post‐infection of people who survived COVID‐19 infection. We employed a random‐effect model for the pooled analysis, and the meta‐analytical effect size was prevalence for the applicable end‐points, *I* ^2^ statistics, and quality assessment of included studies using the Newcastle‐Ottawa Scale. Eleven articles were included after the literature search yielded 223 potentially relevant articles. We found that among patients with long COVID, fatigue, sleep disturbances, and dyspnea were the most common symptoms. Pooled analysis showed that the proportion of individuals experiencing at least one persistent symptom 3 years post‐COVID‐19 is 20% (95% confidence interval [CI]: 8–43). The prevalence of persistent symptoms was dyspnea (12%; 95% CI: 10–15), fatigue (11%; 95% CI: 6–20), insomnia (11%; 95% CI: 2–37), loss of smell (7%; 95% CI: 5–8), loss of taste (7%; 95% CI: 3–16), and anxiety (6%; 95% CI: 1–32). Prevalence of other findings include impaired diffusion capacity (42%; 95% CI: 34–50) and impaired forced expiratory volume in 1 s (10%; 95% CI: 8–12). Our findings confirm the persistence of unresolved symptoms 3 years post‐COVID‐19 infection, with implications for future research, healthcare policy, and patient care.

**Abbreviations**
CI: confidence interval
COVID‐19: coronavirus disease 2019
ICU: intensive care unit
PRISMA: preferred reporting items for systematic review and meta‐analyses
RR: risk ratio
SARS‐CoV‐2: severe acute respiratory syndrome coronavirus 2

## 1 Introduction

Severe acute respiratory syndrome coronavirus 2 (SARS‐CoV‐2) infection can lead to long‐lasting, persistent, and new symptoms occurring after the acute phase of coronavirus disease 2019 (COVID‐19), known as long COVID or post‐acute sequelae of COVID‐19 (PASC) [1, 2, 3, 4, 5, 6, 7, 8, 9, 10, 11]. Similar long‐lasting health effects were also observed in the SARS epidemic in 2003 and at 1 year after hospital discharge, which 18% of patients had a significant reduction in distance walked in 6 min, 17% had not returned to work, and 33% reported a significant decrement in mental health [12]. SARS infection in 2003 also resulted in persistent psychiatric morbidities and chronic fatigue among the survivors for up to 4 years of follow‐up [13]. Even 15 years after SARS epidemic in 2003, 38% of infected patients still had reduced lung diffusion capacity [14]. These reports indicate that long COVID from SARS‐CoV‐2 parallels the long‐term effects seen in the SARS epidemic in 2003, where survivors faced persistent physical and mental health issues for years.

Meta‐analysis studies of long‐term sequelae of COVID‐19 1‐ and 2‐year after SARS‐CoV‐2 infection described risk trajectories for neurological, physical, and psychological sequela and many other outcomes [15, 16, 17]. There are some reports implicating that risks remain in the third year after infection and survivors with long COVID are at increased risk of varying adverse outcomes, such as diabetes, cardiovascular disease, neurological diseases, and kidney disease which might reduce individuals' quality of life and limit daily activity, and resulting in higher unemployment rates [1, 2, 3, 4, 5, 6, 7, 8, 9, 10, 11]. It is not clear to what extent these symptom clusters persist beyond 3 years after infection. Importantly, there is no previous systematic review that described prolonged COVID‐19 symptoms beyond 3 years after SARS‐CoV‐2 infection. Owing to insufficient evidence, we provide a systematic evaluation and detail, which will estimate the pooled prevalence of Long COVID symptoms up to 3 years after SARS‐CoV‐2 infection and also identify the potential risk predictors of these persistent symptoms up to 3 years after infection. Addressing this knowledge gap will facilitate policy development and inform long‐term management strategies in the prevention and response to COVID‐19, and also will inform the care of people with this condition.

## 2 Methods

The present systematic review and meta‐analysis adhered to the methodological guidelines from the Cochrane Handbook for Systematic Reviews [18] and followed the PRISMA (preferred reporting items for systematic review and meta‐analyses) statement 2020 in conducting and reporting the review (Supporting Information S1: Table S1) [19]. The systematic review was pre‐registered with the International Prospective Register of Systematic Reviews (PROSPERO; ref. no. CRD42024581975).

### 2.1 Search Strategy

Two researchers (M.R. and R.U.) electronically searched six databases, including PubMed, MEDLINE (Ovid), CENTRAL, Web of Science, Scopus, and EMBASE from inception of the databases up to July 20, 2024. Any disagreements were resolved through discussion with a third reviewer (L.B.). Search terms were developed through a review of previous systematic reviews on long COVID and relevant literature. Two independent reviewers (M.R. and R.U.) refined the terms through iterative discussion, and a third researcher (L.B.) reviewed the final strategy to ensure clarity and comprehensiveness. The search strategy and terms are provided in Supporting Information S1: Table S2. To find all eligible articles, we searched all reference lists of included studies related to the research question and no language restrictions for studies with English summary were applied in our systematic search.

### 2.2 Eligibility Criteria

The present systematic review and meta‐analysis followed the PICO criteria for inclusion of studies [20]. PICOs: Participants included survivors of COVID‐19; Outcome included those studies reporting on prevalence of long COVID symptoms; Study period describes studies reporting prolonged symptoms present for at least 3 years after initial SARS‐CoV‐2 infection; Intervention and Comparison are not applicable in the present study. We included studies that explicitly reported outcomes at a 3‐year follow‐up after SARS‐CoV‐2 infection. In cases where studies reported multiple follow‐up periods (e.g., 1‐, 2‐, and 3‐year), only the data corresponding to the 3‐year time point were extracted and analyzed. Studies that did not clearly distinguish the outcomes assessed specifically at 3 years, or reported aggregated data across different time points, were excluded to maintain methodological consistency and ensure precision in the synthesis of long‐term outcomes. We also included prospective cohort and cross‐sectional studies. Moreover, we included studies that evaluated the prevalence of long‐term sequelae of COVID‐19 3 years after SARS‐CoV‐2 infection. Additionally, we did not apply any restrictions related to gender or geographical focus. Narrative literature reviews were excluded from the analysis as they do not present original primary data or employ systematic methodology required for meta‐analytic synthesis. However, the reference lists of relevant narrative reviews were screened to identify potentially eligible primary studies not retrieved in the initial search, thereby enhancing the comprehensiveness of the review process. The selection of the articles was independently performed by two reviewers (M.R. and R.U.) at title and abstract screening stage and full‐text report screening stage, and disagreements were resolved through discussion with a third reviewer (L.B.).

### 2.3 Data Extraction and Quality Assessment

We extracted data using Covidence systematic review software (version 2; Veritas Health Innovation, Melbourne, VIC, Australia) on a pre‐designed spreadsheet following Cochrane guidelines. The following data were extracted from the eligible studies: author and year, study design, country, sample size, age, sex, SARS‐CoV‐2 infection period, outcomes, vaccination history, and population characteristics. Outcomes included disability‐adjusted life years, neurologic, mental, fatigue, pulmonary, cardiovascular, musculoskeletal, kidney, gastrointestinal, and metabolic. The quality of included studies was assessed using the Newcastle–Ottawa Scale (NOS). The NOS scores were categorized as follows: scores of 0–3 were considered low quality, scores of 4–6 were considered moderate quality, and scores of 7–9 were considered high quality [21]. Data extraction and quality assessment were independently performed by two reviewers (M.R. and D.K.Y.), and disagreements were resolved through discussion with a third reviewer (L.B.) [22, 23, 24]. Although, had pre‐planned to use a third‐party resolution, no disagreements were present.

### 2.4 Statistical Analyses and Synthesis

Long COVID symptoms 3 years after SARS‐CoV‐2 infection were pooled and expressed as proportions with corresponding 95% confidence intervals (CI). A one‐stage random effects model was employed for the meta‐analysis, as it accounts for both within‐study and between‐study variability, providing more accurate and generalizable estimates of the overall effect. This model is particularly suitable when studies are expected to differ in terms of their designs, populations, and measures of outcome. The degree of between‐study heterogeneity that could not be ascribed to sampling error was explored using Cochran's *Q* statistics and I‐squared (*I* ^2^; low: 0%–40%, moderate: 30%–60%, substantial: 50%–90%, and considerable: > 90%) to estimate heterogeneity in all included studies. Moreover, to assess the robustness of summary estimates and to detect if any study accounted for a large proportion of heterogeneity, sensitivity analysis was performed by the leave‐one‐out method approach. Due to the requirement that Egger's test and Begg's test need at least 10 studies for reliable results, these tests were not performed in the present study, as the number of studies included in each analysis was fewer than 10. All meta‐analyses in the current study were conducted in R software using the “meta” package (version 4.2.2; R Foundation, Vienna, Austria) and a two‐sided *p* < 0.05 was considered significant.

## 3 Result

### 3.1 Study Identification and Characteristics

A total of 223 potentially relevant articles were identified in our literature search. After removing duplicates, 152 articles remained. After screening titles and abstracts, 131 research articles were excluded. From the remaining 21, an additional 10 articles were excluded (Supporting Information S1: Table S3), resulting in 11 articles included in this systematic review [1, 2, 3, 4, 5, 6, 7, 8, 9, 10, 11]. These studies were published between 2023 and 2024.

The included studies were conducted across six countries, including Bulgaria, China (*n* = 5, with four studies from Wuhan), Japan, Italy, Romania, and the United States (*n* = 2). All studies focused on hospitalized COVID‐19 patients from January to December 2020, except for one study that included patients from February 2020 to November 2021. In total, these studies reported data on 142 171 individuals (range 88–135 161) aged 36–86 years with long COVID. Among the 141 847 participants for whom sex or gender data were available, 123 675 (87%) were male and 18 172 (13%) were female. Most were cohort studies (9/11 [81.8%]), followed by cross‐sectional studies (2/11 [18.2%]). Included studies were of medium to high quality, with NOS scores of between 7 and 9 (Supporting Information S1: Table S4 and Supporting Information S1: Figure S1).

### 3.2 Risk of Mortality and Disability‐Adjusted Life Years

Those with COVID‐19 who were not hospitalized and hospitalized during the acute phase of the disease were at an increased risk of death 3 years after SARS‐CoV2 infection (incidence rate ratio [IRR]: 1.01, 95% CI: 0.97–1.04; excess burden per 1000 persons: 0.22, 95% CI: −1.14 to 1.58; and IRR: 1.29, 95% CI: 1.19–1.40; excess burden per 1000 persons: 8.16, 95% CI: 4.37–11.96, respectively) [3]. Among both nonhospitalized and hospitalized people with COVID‐19, long COVID contributed 9.6 (95% CI: 0.4–18.7) and 90.0 (55.2–124.8) disability‐adjusted life years (DALYs) per 1000 persons in the third year, respectively [3]. Further, mortality rates were also higher in both people who had COVID‐19 with and without neurological signs post‐COVID‐19 hospitalization at 3 years (58/414 [14.01%] and 94/1196 [7.84%], respectively) [5]. Overall, these findings highlight the lasting impact of COVID‐19 on mortality and disability up to 3 years after infection, particularly among individuals who required hospitalization and those who experienced neurological complications. The evidence underscores the need for ongoing monitoring, support, and targeted interventions for affected individuals, especially those with severe or complex post‐acute presentations.

### 3.3 Three‐Year Outcomes of Post‐Acute Sequelae of COVID‐19

Although a general reduction in long COVID symptoms among previously hospitalized patients was observed over time, a substantial burden of new or persistent symptoms remained at the 3‐year mark. We categorized these symptoms into eight major physiological clusters: neurologic, mental, pulmonary, cardiovascular, musculoskeletal, kidney, gastrointestinal, and metabolic (Supporting Information S1: Table S5). Among these, the neurologic, pulmonary, and cardiovascular clusters were the most commonly affected. Notably, neurological symptoms such as memory problems, dizziness, and peripheral neuropathy were frequent, along with persistent fatigue, mental health issues including anxiety and depression, pulmonary impairments like dyspnea and reduced lung function, and cardiovascular complications including heart failure and arrhythmias [1, 2, 3, 4, 5, 6, 7, 8, 9, 10, 11]. For a detailed list of all reported symptoms, please refer to Supporting Information S1: Table S5.

### 3.4 Identified Risk Factors Associated With the Persistence of Post‐Acute Sequelae of COVID‐19

**Figure 1:**
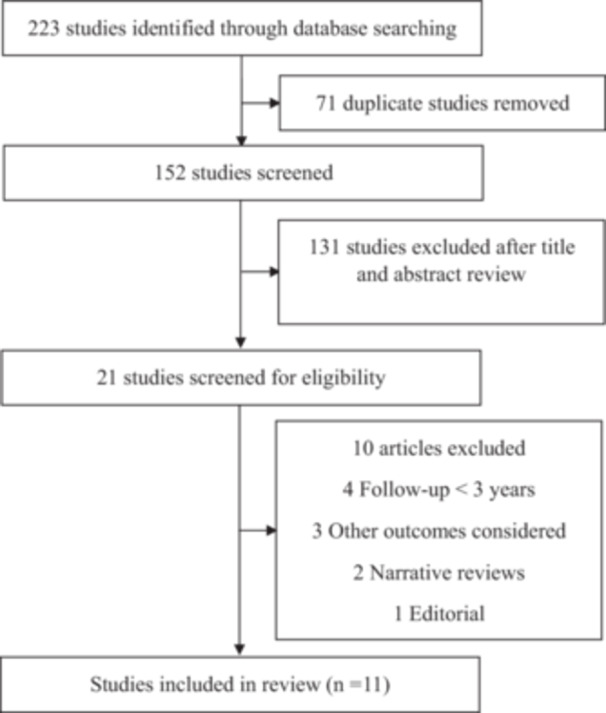
PRISMA flow diagram of study selection.

To help clinicians, public health policy makers, and researchers efficiently identify COVID‐19 survivors most at risk of long‐term sequelae 3 years after SARS‐CoV2 infection the present review identified the key risk factors in the literature for long COVID and these included older age (possibly due to immunosenescence and pre‐existing vulnerabilities) [5, 11], higher COVID‐19 severity score at hospitalization (reflecting greater initial organ damage) [2, 3, 4, 5, 7, 9, 11], female sex (potentially linked to sex‐based immunological differences) [7, 10, 11], smoking [2, 11], substance use [2], allergy [9], and having comorbidities (including congestive heart failure, chronic kidney disease, hypertension, and diabetes) [5, 6, 9]. Further, consuming Renin–angiotensin–aldosterone blockers before and during the pandemic, had no discernible effect during the postinfection period on reported long COVID symptoms [6] (Figure 1).

### 3.5 Meta‐Analysis of Post‐Acute Sequelae of COVID‐19

**Figure 2:**
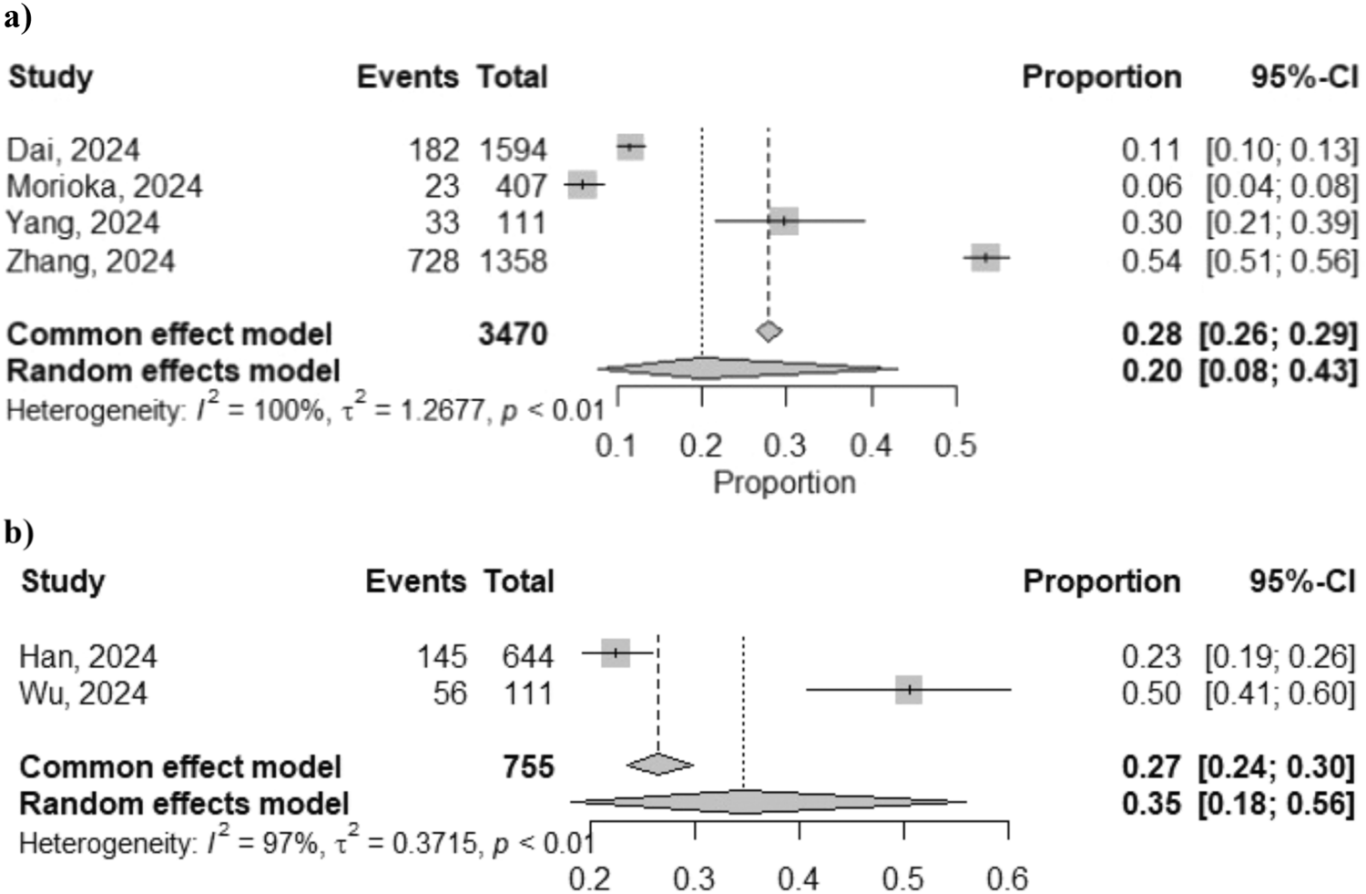
Forest plot of (a) at least one unresolved symptom and (b) at least one unresolved respiratory symptom 3 years after SARS‐CoV‐2 infection.

The pooled prevalence of COVID‐19 survivors experiencing at least one unresolved symptom and at least one unresolved respiratory symptom 3‐years after SARS‐CoV‐2 infection, were 20% (95% CI: 8%–43%; *Z* = 2.22, *p* = 0.01; four studies [4, 10, 11]) and 35% (95% CI: 18%–56%; *Z* = 2.59, *p* = 0.009; two studies [7, 9]), respectively (Figure 2).

The most frequent significant findings at 3‐years after SARS‐CoV‐2 infection were DLCO < 80% of the predicted value (42%; 95% CI: 34%–50%; *Z* = 7.46, *p* = 0.0001; two studies [7, 9]) and forced expiratory volume in 1 s (FEV1) < 80% of the predicted value (10%; 95% CI: 8%–12%; *Z* = 8.85, *p* = 0.0001; two studies [7, 9]). The most frequent significant symptoms at 3 years following SARS‐CoV‐2 were dyspnea (12%; 95% CI: 10%–15%; *Z* = 7, *p* = 0.0001; two studies [7, 9]), fatigue (11%; 95% CI 6%–20%; *Z* = 3.23, *p* = 0.001; three studies [4, 10, 11]), smell disorder (7%; 95% CI 5%–8%; *Z* = 3.42, *p* = 0.0006; two studies [1, 11]), cough (6%; 95% CI: 2%–15%; *Z* = 2.35, *p* = 0.018; two studies [4, 7, 9, 11]), and expectoration (4%; 95% CI: 1%–11%; *Z* = 2.19, *p* = 0.028; three studies [4, 7, 9]). The prevalence of sleep difficulty (11%; 95% CI: 2%–37%; *Z* = 1.30, *p* = 0.1; two studies [10, 11]), taste disorder (7%; 95% CI: 3%–16%; *Z* = 1.64, *p* = 0.1; two studies [1, 11]), anxiety (6%; 95% CI: 1%–32%; *Z* = 1.14, *p* = 0.255; two studies [4, 11]), and myalgia (4%; 95% CI: 2%–18%; *Z* = 1.89, *p* = 0.058; two studies [4, 11]) were not significant (Figure 3).

Figure 3Forest plot of prevalence of various symptoms 3 years after SARS‐CoV‐2 infection.

**Figure:**
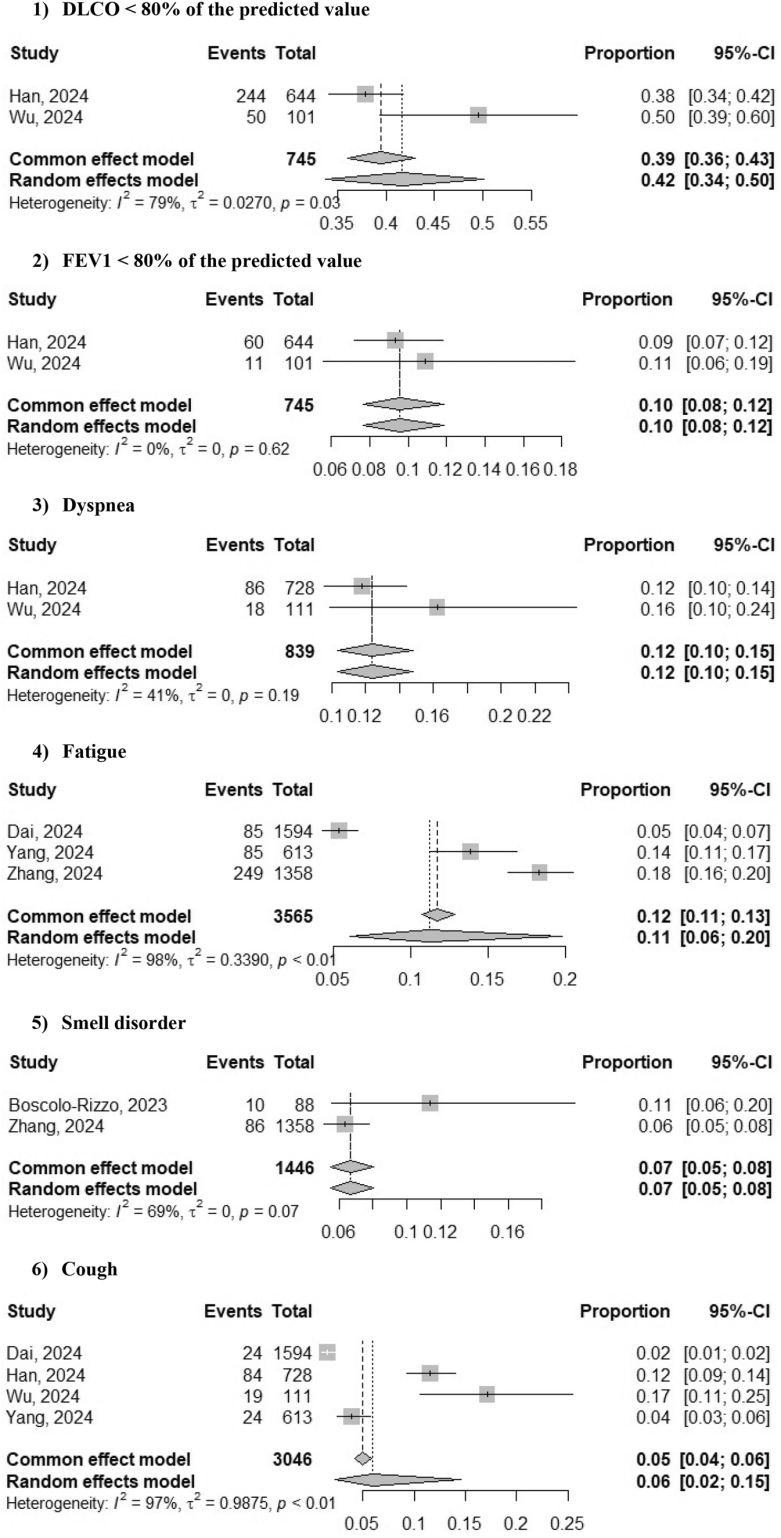

**Figure:**
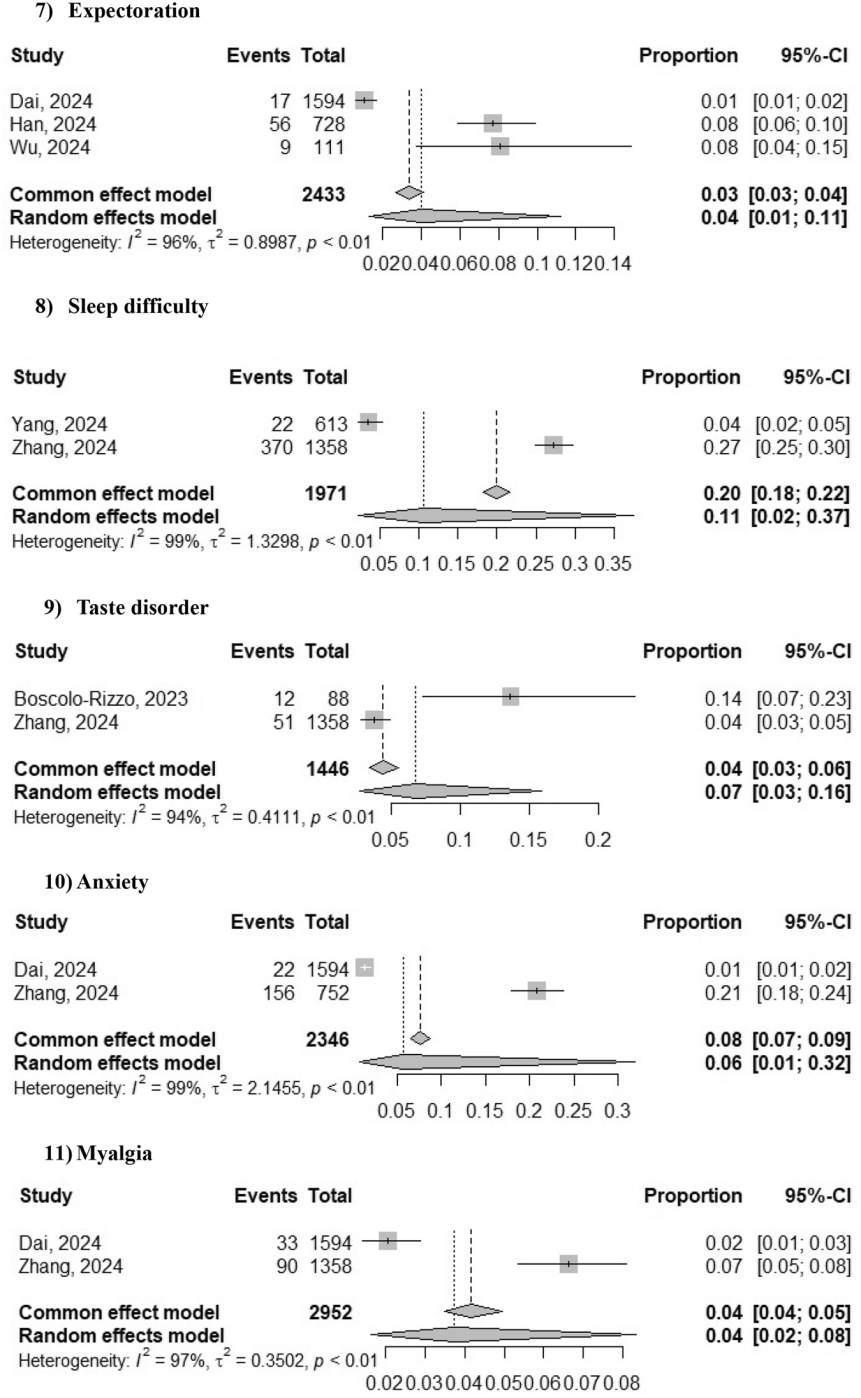

## 4 Discussion

This study signifies the first systematic review and meta‐analysis focused on the post‐acute sequelae of COVID‐19 over a 3‐year period. Evidence from 11 distinct studies was synthesized to examine the mental, neurological, and physical symptoms experienced by survivors of COVID‐19. The key finding is that the pooled prevalence of survivors of COVID‐19 experiencing at least one unresolved symptom after 3 years of initial SARS‐CoV‐2 infection is 20%. The most prevalent unresolved symptoms 3 years after SARS‐CoV‐2 infection were dyspnea (12%), fatigue (11%), and insomnia (11%). Other symptoms include smell (7%) and taste (7%) disorders, anxiety (6%), cough (6%), expectoration (4%), and myalgia (4%). Notably, the most prevalent findings impaired diffusion capacity (42%) as well as impaired forced expiratory volume in 1 s (10%). This empirical evidence consists mostly of data on patients with long COVID who were hospitalized during the acute phase of the illness, which suggest caution in generalizing our findings for patients with long COVID who had mild symptoms during the initial infection.

This review found that dyspnea, fatigue, and sleep problems were the most prevalent post‐COVID symptoms 3‐years after infection with a prevalence range of 11%–12%. Dyspnea was the most frequent symptom. Though dyspnea showed a relatively slight increase from 10% (2‐year prevalence) to 12% (present study), its pulmonary assessment showed no significant difference with the 2‐year prevalence report on lung diffusion capacity and other lung function tests [17]. Patients with long COVID in the Wuhan cohort that completed lung function test at 3 years following hospital discharge showed dysfunctional residual volume and forced expiratory volume, as well as impaired lung diffusion capacity [9, 11]. Fatigue is the second most frequent symptom, and it shows a substantial reduction from the reported 2‐year follow‐up data of 27.4% (15) and 28% (21) to 11% in the present study. This agrees with Zhang et al., who also reported a significant reduction in fatigue prevalence [11]. Possible explanations for that include some therapeutic measures that could have over time reduced fatigue or raised physical activity following termination of all restrictions post‐COVID‐19 or people have adapted better over time [2]. Other problems reported at 3 years following hospital discharge from COVID‐19 include the following: cardiovascular (angina, bradycardia, and cardiac arrest, etc), musculoskeletal (myalgia, joint pain, and osteoarthritis), kidneys (acute kidney injury and kidney failure) as well as diabetes.

Han et al. had previously reported that the prevalence rate of most symptoms of PASC within the first year following initial infection to be substantial [25]. Two years after initial infection, Rahmati and colleagues showed that 42% of survivors of COVID‐19 still experienced at least one unresolved symptom of PASC [15]. Current evidence provides data that suggests the prevalence of long COVID symptomatology reduces over time with few exceptions. A well‐designed meta‐analysis which pooled data from 54 included studies across over 20 nations showed that fatigue, sleep difficulties, and dyspnea are the most frequent symptom cluster in the first 3 months following initial SARS‐CoV‐2 infection [26].

This review reports that there's great improvement in the major neuropsychiatric symptoms of fatigue, sleep disorders, and anxiety 3 years post‐COVID‐19—following a consistent decline over time. However, a worsening trend was observed for the other neurological symptoms such as smell and taste loss. At 3 years, the pooled prevalence of sleep disorders, anxiety, smell and taste problems were 11%, 6%, 7%, and 7%, respectively; compared to their 2‐year pooled prevalence of 25%, 9%, 3.5%, and 4.5%, respectively [17]. However, longer‐term follow‐up will certainly establish any definitive trend of symptom improvement. Other neuropsychiatric symptoms reported at 3 years include memory problems, Parkinson‐like disease, alcohol use disorder, peripheral neuropathy, and vision impairment [1, 3, 4, 11]. However, a longer‐term study will be required to explore an association between cognitive dysfunction (often called brain fog) and neurodegenerative disorder following COVID‐19 [27].

Similar coronavirus infections such as SARS and MERS have been known to cause long‐term chronic effects such as fatigue, sleep disorders, anxiety disorders, and lung damage. The longest documented longitudinal study post‐SARS infection is by Li and et al. [28]. The study which followed up and characterized 14 healthcare workers (HCWs) for 18 years postdischarge from SARS infection reported fatigue as the most common symptomatic complaint, followed by dyspnea despite normal pulmonary and gas diffusion capacity in the face of persistent lung lesions up to 18 years, as well as fully recovered mental and emotional health [28]. Li et al. further reported that when the 18‐year recovery was compared to their 12‐year recovery cohort, the later showed lower physical, social, and emotional functions which significantly improved at 18 years follow‐up, but was still worse than that of the control group [28]. This is consistent with the Peking study, which followed up 71 HCWs for 15 years from Peking University People's Hospital that suffered nosocomial infection during the 2003 SARS outbreak showed that the proportion of patients with lung lesions on CT reduced significantly from 9.4% in 2003 to 3.2% in 2004 and remained stable 15 years later 4.64% [14]. Another study which followed up 11 SARS survivors reported that 10 patients had fibrosis 7 years later compared to 11 patients who had it 6 months post‐discharge [29]. These are in agreement with the work of Han et al., who reported that over 30% of its longitudinal cohort continued to have unresolved pulmonary lesions up to 3 years and they were associated with respiratory symptoms as well as impaired diffusion capacity [7].

A recent meta‐analysis that reviewed 19 studies from 14 different nations regarding work ability and return‐to‐work by patients with long COVID found that unresolved symptoms have a substantial impact on their work‐related activities with 61% returning to work effectively after 3 months or more post‐COVID‐19 [30]. They further reported that an ample proportion of these returnees still require some adjustments to their work duties or number of hours to assist with their unresolved disabilities [30]. According to the US Centre for Disease Control (CDC), a national survey of noninstitutionalized United States adults taken between 2022 and 2023 to estimate long COVID prevalence revealed that 26% of patients with long COVID experienced significant limitation to their daily activity relative to the pre‐COVID period [31]. We are unable to report on the pooled prevalence regarding return to work due to lack of adequate data. The need for researchers to report on the proportion of patients with long COVID who have been able to return to their original job 3 years later cannot be overemphasized. It's important to note that whereas we had two studies report on this parameter during the 2‐year review [17], only one study reported it in the current review [11]. Given that long COVID posse not just a risk of unresolved symptoms but for those with disabilities, living with long COVID can be an additional burden, thus this demands a more comprehensive care approach that addresses the consequential health inequities.

Significant limitation to functional and work capacity is best exemplified by the work of Eligulashvili and colleagues who revealed that half and one‐third of the neurological cohort and non‐neurological cohort, respectively, were discharged to both skilled nursing facilities and rehabilitation homes [5]. Furthermore, the study which followed up 414 patients with SARS‐CoV‐2 infection and substantial neurological findings (e.g., acute stroke, new seizures) versus 1199 COVID‐19 positive patients without substantial neurological findings showed that the neurological cohort were at greater risk of life‐threatening worse longer term sequelae including higher mortality risk after discharge (from heart failure, infections, and ARDS), neurodegenerative diseases, higher readmission risk and higher age‐adjusted brain volume loss [5]. Interestingly, this is strongly supported by the work of Ziyad Al‐Aly and his team which followed up 135 161 COVID‐19 survivors versus 5 206 835 controls from the United States Veterans affairs department to compare their risk of PASC and death [3]. They found that the nonhospitalized patients had no increased risk of death after the first year and a lower risk of PASC up to the third year, while the hospitalized patients had both increased risk of death and significant PASC risk up to the third year post‐COVID‐19 [3].

Recent studies have identified several mechanisms contributing to long COVID symptoms. One significant factor is the severity of the acute infection, with more severe cases linked to a higher risk of long‐term symptoms, suggesting greater organ damage and emphasizing the need for early clinical management, especially in high‐risk patients [32, 33]. Another key factor is the generation of autoantibodies, which can trigger inflammation and tissue damage, explaining persistent symptoms such as fatigue, neurological dysfunction, and cardiovascular issues [34]. Lastly, the persistence of SARS‐CoV‐2 RNA in tissues may lead to ongoing immune activation and inflammation, contributing to chronic symptoms [35]. These mechanisms offer insights into long COVID's pathogenesis and potential treatment strategies, although further research is necessary to fully understand their interactions and long‐term effects.

Despite our extensive literature search that explored multiple databases, a key limitation of this review is the availability of data on persistent symptoms in hospitalized patients with little or no data regarding residual symptoms in patients with a mild course of COVID‐19. Our data pooling capacity in subgroup analysis was further limited by studies with a mixed cohort of hospitalized and mild cases, as well as little information regarding data about patients requiring ICU admission. Another limitation was the use of qualitative studies as we endeavored to provide a more comprehensive illumination into the experience of patients with long COVID 3 years after the initial SARS‐CoV‐2 infection. Also, the use of self‐reported questionnaires has the risk of introducing reporting bias.

The small number of studies in each analysis reduces statistical power and precision. Additionally, substantial heterogeneity in some comparisons weakens confidence in the generalizability of the results. This variability may be influenced by factors such as viral variants, population demographics, vaccination rates, treatment protocols, and reinfections, all of which could contribute to inconsistent findings. For symptoms such as insomnia and anxiety, the wide CIs and high heterogeneity suggest considerable variability between studies, and as a result, the pooled estimates should be interpreted with caution. Furthermore, the lack of individual‐level data on symptom co‐occurrence (e.g., dyspnea and fatigue) limits the ability to assess symptom overlap across cases, restricting our ability to explore symptom clustering and potential interaction effects. Another important limitation is the heterogeneity in the definition of control groups across the included studies. Some studies used healthy individuals with no prior SARS‐CoV‐2 infection as controls, while others included individuals who had recovered from acute COVID‐19 without developing long COVID symptoms. Due to the limited number of studies, subgroup analyses based on control group type could not be conducted. This heterogeneity may have influenced the pooled estimates and should be taken into consideration when interpreting the findings.

The included studies did not consistently report on SARS‐CoV‐2 variants, limiting our ability to assess the impact of variant differences on post‐COVID symptoms. Previous research suggests that different variants may affect clinical outcomes, highlighting the need for variant‐specific data in long‐term studies [36]. Future research should investigate the role of variants, reinfections, and psychosocial factors in long COVID development and recovery, helping to identify predictors of symptom persistence and alleviating patients' concerns about the duration of their condition. Efforts should focus on mechanistic research for long COVID, biomarker discovery, and comparing long COVID to other chronic infection‐related conditions (e.g., SARS, influenza, MERS, and Ebola). Future research should also include diverse populations, such as those with mild acute cases or underlying conditions like diabetes. Additionally, investigating the long‐term trajectory of long COVID, particularly the association between cognitive issues (e.g., brain fog) and the risk of neurodegenerative disorders, is crucial. Despite a decline in long COVID prevalence, its impact on health‐related quality of life, functional capacity, and socioeconomic consequences, along with the strain on public health systems, remains significant. These findings emphasize the need for public health policies focusing on prevention, such as improving COVID vaccination effectiveness and promoting early antiviral use during the acute phase [24, 37, 38]; and could potentially inform the formulation of disability policy, health care service needs and other rehabilitative services for patients with long COVID who have limited functional and work capacity.

## 5 Conclusion

The long‐term physical and neuropsychiatric consequences of long COVID persist as a significant clinical challenge for many survivors, even up to 3 years after initial infection. This study enhances our understanding of the natural trajectory of recovery and underscores the urgent need for comprehensive, sustained care strategies. Despite some improvement in symptoms, the management of long COVID remains hindered by several systemic issues: limited clinical guidance, insufficient diagnostic and treatment protocols, and fragmented care approaches. Additionally, delays in recognition and misattributions of symptoms to psychosomatic causes may compromise patient outcomes and trust. Addressing these challenges requires a multi‐faceted response. Healthcare professionals must be supported through ongoing specialized training and evidence‐based clinical guidelines informed by the latest research. Establishing multidisciplinary care teams and integrating long COVID management into routine healthcare pathways are essential steps toward improved care delivery. Moreover, public health campaigns are vital to raise awareness, reduce stigma, and foster informed patient engagement. By improving knowledge among the public and practitioners alike, we can build a more responsive and empathetic healthcare system. Ultimately, tackling long COVID must be recognized as a public health priority. Coordinated global action, informed by robust evidence and compassionate care, will be key to supporting the recovery and long‐term well‐being of those affected (Tables 1 and 2).

**Table 1:** General characteristics of included studies.

Study	Design	Country	Number	Mean age (SD)	Female sex, *n* (%)	SARS‐CoV‐2 infection period	Outcome evaluation	Study population characteristics
Boscolo‐Rizzo et al. [1]	Cohort	Italy	88	49 (10)	51 (58)	March and April, 2020	Olfactory (Sniffin' Sticks test battery) and gustatory (Taste Strips test) function	Mild patient who had symptoms of COVID‐19 without evidence of lower respiratory disease during clinical assessment or imaging and oxygen saturation of 94% or greater
Bota et al. [2]	Cross‐sectional	Romania	324	56 (8)	NR	2020	Depression (PHQ‐9), anxiety (GAD‐7), and quality of life (SF‐36)	Hospitalized patients with mild to moderate COVID‐19 who had surveyed 6 months after their hospitalization for SARS‐CoV‐2 infection
Cai et al. [3]	Cohort	USA	135 161	64 (17)	14 987 (11)	March and December, 2020	80 individual outcomes based on ICD‐10 codes and well‐characterized sequelae of SARS‐CoV‐2 infection	Patients with COVID‐19 who were alive at 30 days after the infection and divided by the care setting during the acute phase of infection into nonhospitalized and hospitalized (inpatient admission date within 7 days before or within 30 days after the positive test)
Dai et al. [4]	Cohort	China	1594	58 (9)	798 (50)	February and April, 2020	Prevalence of at least one PASC symptoms, symptom relief, and new‐onset symptom	Patients with COVID‐19 who were discharged from Huoshenshan Hospital and Taikang Tongji Hospital in Wuhan, China
Eligulashvili et al. [5]	Cohort	USA	1613	70 (15)	764 (47)	March and April, 2020	Mortality, stroke, heart attack, MACE, reinfection, and hospital readmission post‐discharge	Hospitalized patients with COVID‐19 who experienced various neurological signs and symptoms that warranted neuroimaging during COVID‐19 hospitalization were compared with patients with COVID‐19 who did not have significant neurological issues during hospitalization
Filev et al. [6]	Cohort	Bulgaria	120	61 (15)	60 (50)	February and March, 2021	Renal function	Hospitalized patients with COVID‐19 and chronic kidney disease were compared with patients with COVID‐19 and without chronic kidney disease and also with healthy control group without history of SARS‐CoV2 infection
Han et al. [7]	Cohort	China	781	61 (8)	310 (43)	January and April, 2020	Prevalence of at least one respiratory symptom, symptom persist, respiratory symptoms, and lung function	Patients with COVID‐19 who were discharged from Jin Yin‐tan Hospital and Wuhan Union Hospital in Wuhan, China
Morioka et al. [8]	Cross‐sectional	Japan	407	48 (6)	253 (58)	February 2020 to November 2021	Prevalence of at least one PASC symptoms	Patients who had recovered from COVID‐19. 99% of included patient had mild to moderate COVID‐19 severity
Wu et al. [9]	Cohort	China	111	64 (5)	42 (38)	January and April, 2020	Prevalence of at least one respiratory symptom, respiratory symptoms, and lung function	Patients with diabetes and COVID‐19 who were discharged from two major tertiary hospitals (center 1 and center 2) in Wuhan, China
Yang et al. [10]	Cohort	China	613	62 (9)	276 (45)	January and May, 2020	Prevalence of at least one PASC symptoms, symptom persist, respiratory symptoms, and lung function	Patients with COVID‐19 who were discharged in accordance with the Chinese clinical guidelines for COVID‐19 pneumonia diagnosis and treatment
Zhang et al. [11]	Cohort	China	1359	57 (8)	631 (46)	January and May, 2020	Prevalence of at least one PASC symptoms, 20 individual outcomes, Depression (PHQ‐9), anxiety (GAD‐7), quality of life (EQ‐5D‐5L), and posttraumatic stress disorder symptom (PCL‐C)	Patients with COVID‐19 who were discharged from the Jin Yin‐tan hospital in Wuhan, China

Abbreviations: GAD‐7, generalized anxiety disorder 7‐item scale; NR, not reported; PHQ‐9, patient health questionnaire‐9.

**Table 2:** Clinical presentation, symptoms, and factors associated with long COVID.

Study	Follow‐up, year	Clinical presentation and symptoms, *n*/*N* (%)	Factors associated with the persistence of PASC
Boscolo‐Rizzo et al. [1]	1 year, 2 years, 3 years	Neurologic: olfactory dysfunction (12/88 [13.6%]), Gustatory dysfunction (10/88 [11.4%])	NR
Bota et al. [2]	1 year, 2 years, 3 years	Psychiatric: depression (NR), anxiety (NR), and quality of life (NR)	Smoking, substance use, and severity of COVID‐19 at hospitalization, were associated with depression
Cai et al. [3]	1 year, 2 years, 3 years	Incidence rate ratio, number of sequelae and disability‐adjusted life years (DALYs) due to COVID‐19 in nonhospitalized and hospitalized patients with COVID‐19 in 3 years after SARS‐CoV‐2 infection were mentioned for different organs and symptoms including: cardiovascular, coagulation, fatigue, gastrointestinal, kidney, mental, metabolic, musculoskeletal, neurologic, and pulmonary	Patients with COVID‐19 who were hospitalized during the acute phase of SARS‐CoV‐2 infection had significantly higher risk and burden of overall PASC and sequelae in every organ system
Dai et al. [4]	1 year, 2 years, 3 years	11.4% (182/1594) proportions of participants had at least one symptom at 3 years after symptom onset or COVID‐19 diagnosis; Neurologic: fatigue (85/1594 [5.3%]) and myalgia (33/1594 [2.1%]); Respiratory: cough (24/1594 [1.5%]), shortness of breath (19/1594 [1.2%]), and expectoration (17/1594 [1.1%]); Cardiac: chest tightness (32/1594 [2%]); Psychiatric: anxiety (22/1594 [1.4%])	Multivariate logistic regression analysis showed that mechanical ventilation at hospitalization was associated with presence of at least one symptom. Univariable analysis showed that ICU admission at hospitalization was associated with higher risk of fatigue and symptom persistence.
Eligulashvili et al. [5]	1 year, 2 years, 3 years	Patients with neurological signs: stroke (26/414 [6.28%]), major adverse cardiovascular events (85/414 [20.53%]), and mortality (58/414 [14.01%]) Patients without neurological signs: stroke (28/1196 [2.34%]), major adverse cardiovascular events (198/1196 [16.51%]), and mortality (94/1196 [7.84%])	Among both patients with and without neurological signs, non‐survivors were significantly older and had higher COVID‐19 severity score at hospitalization than survivors. In patients without neurological signs, non‐survivors had higher incidence of hypertension, diabetes, congestive heart failure, and chronic kidney disease compared to control survivors. Univariate logistic regression analysis showed that congestive heart failure, COVID‐19 severity score, and age were associated with mortality in patients with neurological signs.
Filev et al. [6]	3 year	Creatinine and proteinuria or albumin/creatinine ratio significantly improved 3 years after the infection with patients without chronic kidney disease showing better recovery of renal function than those with any stage of chronic kidney disease before the COVID‐19 infection	Logistic regression analysis showed that consuming Renin–angiotensin–aldosterone blockers before and during the pandemic, had no discernible effect during the postinfection period on reported PASC symptoms, including fatigue, cognitive dysfunction, chest pain, and sleep disturbances
Han et al. [7]	1 year, 2 years, 3 years	23% (145/644) proportions of participants had at least one respiratory symptom at 3 years after symptom onset or COVID‐19 diagnosis; Respiratory: cough (84/728 [12%]), dyspnea (86/728 [12%]), expectoration (56/728 [7.7%]) Pulmonary function: FEV1 < 80% predicted (40/644 [6.2%]), DLCO < 80% predicted (244/644 [38%])	Multivariate logistic regression analysis showed that higher COVID‐19 severity score at hospitalization and female gender were associated with higher risks of lung diffusion impairment
Morioka et al. [8]	1 year, 2 years, 3 years	5.7% (4/70) proportions of participants had at least one symptom at 3 years after symptom onset or COVID‐19 diagnosis	NR
Wu et al. [9]	1 year, 2 years, 3 years	50.5% (56/111) proportions of participants had at least one respiratory symptom at 3 years after symptom onset or COVID‐19 diagnosis; Respiratory: cough (19/111 [17.1%]), dyspnea (18/111 [16.2%]), expectoration (9/111 [8.1%]) Pulmonary function: FEV1 < 80% predicted (11/101 [10.9%]), DLCO < 80% predicted (50/101 [49.5%])	Multivariate logistic regression analysis showed that higher COVID‐19 severity score at hospitalization and female gender were associated with higher risks of lung diffusion impairment
Yang et al. [10]	2 years, 3 years	29.7% (33/111) proportions of participants had at least one symptom at 3 years after symptom onset or COVID‐19 diagnosis; Neurologic: fatigue (85/613 [17%]); Respiratory: cough (24/613 [3.8%]), shortness of breath (19/613 [5.3%]); Psychiatric: difficulty sleeping (22/613 [9.8%]), joint pain (22/613 [6.9%])	Multivariate logistic regression analysis showed that diabetes, allergy, severe COVID‐19, and female gender were associated with impaired independent predictors of pulmonary diffusion dysfunction
Zhang et al. [11]	2 years, 3 years	54% (728/1358) proportions of participants had at least one symptom at 3 years after symptom onset or COVID‐19 diagnosis. Neurologic: fatigue (249/1358 [18%]), sleep difficulties (370/1358 [27%]), smell disorder (86/1358 [6%]), taste disorder (51/1358 [4%]), dizziness (77/1358 [6%]), myalgia (90/1358 [7%]), headache (61/1358 [4%]). Cardiac: palpitation (123/1358 [9%]). Psychiatric: depression (278/752 [37%]), anxiety (156/752 [21%]), pain or discomfort (393/752 [52%]), mobility problem (33/752 [4%]), usual activity problem (12/752 [2%]), and posttraumatic stress disorder symptom (120/16 [52%])	Multivariate logistic regression analysis showed that higher COVID‐19 severity score at hospitalization and older age were associated with dyspnea. Older age was associated with reduced daily activity. Female gender was associated with higher dyspnea and anxiety or depression symptoms and lower EQ‐VAS score. Regarding the vaccination status, administrating three or more doses of COVID‐19 vaccines was positively associated with EQ‐VAS score and negatively with reduced daily activity. Smoking was associated with depression and lower quality of life scores.

Abbreviations: ADHD, attention‐deficit/hyperactivity disorder; BCVA, best‐corrected visual acuity; NR, not reported.

## Author Contributions

**Masoud Rahmati:** literature search, study selection and data extraction, design, concept, wrote the initial draft, performed statistical analyses. **Raphael Udeh:** literature search, study selection and data extraction, design, concept, wrote the initial draft, performed statistical analyses. **Jiseung Kang:** literature search, study selection and data extraction. **Xenia Dolja‐Gore:** literature search, performed statistical analyses. **Mark McEvoy:** literature search, design, wrote the initial draft, performed statistical analyses. **Abdolreza Kazemi:** study selection and data extraction, wrote the initial draft. **Pinar Soysal:** study selection and data extraction. **Lee Smith:** wrote the initial draft. **Guillaume Fond:** design. **Dong Keon Yon:** literature search, design, wrote the initial draft, performed statistical analyses. **Laurent Boyer:** study selection and data extraction, design, wrote the initial draft. **Masoud Rahmati, Raphael Udeh, Laurent Boyer**, and **Dong Keon Yon** had full access to all data in this study and take responsibility for the integrity of the data and the accuracy of the data analysis. All authors: Critical revision of the manuscript for important intellectual content, reviewed and approved the final manuscript.

## Conflicts of Interest

The authors declare no conflicts of interest.

## Supporting information

Supplementary materials.

## Data Availability

The data that support the findings of this study are available from the corresponding author upon reasonable request. All data relevant to the study are included in the article or uploaded as supporting information. The data are available by accessing the published studies listed in Table 1.
