# Supplementary material for: Long‐Term Sequelae of COVID‐19: A Systematic Review and Meta‐Analysis of Symptoms 3 Years Post‐SARS‐CoV‐2 Infection
Source: J Med Virol. 2025 Jun 6;97(6):e70429. doi: 10.1002/jmv.70429 (PMC12143191; doi:10.1002/jmv.70429)

**Long‐term sequelae of COVID‐19 3‐year after SARS‐CoV‐2 infection: A systematic review**

**Supplementary Materials**

**Contents**

[**Table 1.** PRISMA 2020 Checklist 2](#_Toc196380355)

[**Table 2.** Literature search strategy 5](#_Toc196380356)

[**Table 3.** A list of the excluded studies and reasons for their exclusion 6](#_Toc196380357)

[**Table 4.** Quality assessment and publication bias evaluation of included study using the Newcastle-Ottawa Scale (NOS). 7](#_Toc196380358)

[**Table 5.** Prevalence of different outcomes 3 years after SARS-CoV-2 infection. 8](#_Toc196380359)

[**Figure 1.** Full summary plot for the Newcastle-Ottawa Scale. 11](#_Toc196380360)

# **Table 1.** PRISMA 2020 Checklist

| **Section and Topic** | **Item #** | **Checklist item** | **Location where item is reported** |
| --- | --- | --- | --- |
| **TITLE** | | |  |
| Title | 1 | Identify the report as a systematic review. | 1 |
| **ABSTRACT** | | |  |
| Abstract | 2 | See the PRISMA 2020 for Abstracts checklist. | 3-4 |
| **INTRODUCTION** | | |  |
| Rationale | 3 | Describe the rationale for the review in the context of existing knowledge. | 5 |
| Objectives | 4 | Provide an explicit statement of the objective(s) or question(s) the review addresses. | 5-6 |
| **METHODS** | | |  |
| Eligibility criteria | 5 | Specify the inclusion and exclusion criteria for the review and how studies were grouped for the syntheses. | 7 |
| Information sources | 6 | Specify all databases, registers, websites, organisations, reference lists and other sources searched or consulted to identify studies. Specify the date when each source was last searched or consulted. | 7 |
| Search strategy | 7 | Present the full search strategies for all databases, registers and websites, including any filters and limits used. | 7 |
| Selection process | 8 | Specify the methods used to decide whether a study met the inclusion criteria of the review, including how many reviewers screened each record and each report retrieved, whether they worked independently, and if applicable, details of automation tools used in the process. | 7-8 |
| Data collection process | 9 | Specify the methods used to collect data from reports, including how many reviewers collected data from each report, whether they worked independently, any processes for obtaining or confirming data from study investigators, and if applicable, details of automation tools used in the process. | 7-8 |
| Data items | 10a | List and define all outcomes for which data were sought. Specify whether all results that were compatible with each outcome domain in each study were sought (e.g. for all measures, time points, analyses), and if not, the methods used to decide which results to collect. | 8-9 |
|  | 10b | List and define all other variables for which data were sought (e.g. participant and intervention characteristics, funding sources). Describe any assumptions made about any missing or unclear information. | 8-9 |
| Study risk of bias assessment | 11 | Specify the methods used to assess risk of bias in the included studies, including details of the tool(s) used, how many reviewers assessed each study and whether they worked independently, and if applicable, details of automation tools used in the process. | 8-9 |
| Effect measures | 12 | Specify for each outcome the effect measure(s) (e.g. risk ratio, mean difference) used in the synthesis or presentation of results. | 9 |
| Synthesis methods | 13a | Describe the processes used to decide which studies were eligible for each synthesis (e.g. tabulating the study intervention characteristics and comparing against the planned groups for each synthesis (item #5)). | 7-9 |
|  | 13b | Describe any methods required to prepare the data for presentation or synthesis, such as handling of missing summary statistics, or data conversions. | N/A |
|  | 13c | Describe any methods used to tabulate or visually display results of individual studies and syntheses. | 8-9 |
|  | 13d | Describe any methods used to synthesize results and provide a rationale for the choice(s). If meta-analysis was performed, describe the model(s), method(s) to identify the presence and extent of statistical heterogeneity, and software package(s) used. | 8-9 |
|  | 13e | Describe any methods used to explore possible causes of heterogeneity among study results (e.g. subgroup analysis, meta-regression). | 8 |
|  | 13f | Describe any sensitivity analyses conducted to assess robustness of the synthesized results. | 8 |
| Reporting bias assessment | 14 | Describe any methods used to assess risk of bias due to missing results in a synthesis (arising from reporting biases). | N/A |
| Certainty assessment | 15 | Describe any methods used to assess certainty (or confidence) in the body of evidence for an outcome. | N/A |
| **RESULTS** | | |  |
| Study selection | 16a | Describe the results of the search and selection process, from the number of records identified in the search to the number of studies included in the review, ideally using a flow diagram. | Figure 1 |
|  | 16b | Cite studies that might appear to meet the inclusion criteria, but which were excluded, and explain why they were excluded. | Figure 1 |
| Study characteristics | 17 | Cite each included study and present its characteristics. | Table 1 |
| Risk of bias in studies | 18 | Present assessments of risk of bias for each included study. | Table S4 |
| Results of individual studies | 19 | For all outcomes, present, for each study: (a) summary statistics for each group (where appropriate) and (b) an effect estimate and its precision (e.g. confidence/credible interval), ideally using structured tables or plots. | Figures 2-8,  Figures S1-S14 |
| Results of syntheses | 20a | For each synthesis, briefly summarise the characteristics and risk of bias among contributing studies. | 16 |
|  | 20b | Present results of all statistical syntheses conducted. If meta-analysis was done, present for each the summary estimate and its precision (e.g. confidence/credible interval) and measures of statistical heterogeneity. If comparing groups, describe the direction of the effect. | 10-16 |
|  | 20c | Present results of all investigations of possible causes of heterogeneity among study results. | 10-16 |
|  | 20d | Present results of all sensitivity analyses conducted to assess the robustness of the synthesized results. | 11 |
| Reporting biases | 21 | Present assessments of risk of bias due to missing results (arising from reporting biases) for each synthesis assessed. | Table S4 |
| Certainty of evidence | 22 | Present assessments of certainty (or confidence) in the body of evidence for each outcome assessed. | N/A |
| **DISCUSSION** | | |  |
| Discussion | 23a | Provide a general interpretation of the results in the context of other evidence. | 17-20 |
|  | 23b | Discuss any limitations of the evidence included in the review. | 20-21 |
|  | 23c | Discuss any limitations of the review processes used. | 20-21 |
|  | 23d | Discuss implications of the results for practice, policy, and future research. | 21 |
| **OTHER INFORMATION** | | |  |
| Registration and protocol | 24a | Provide registration information for the review, including register name and registration number, or state that the review was not registered. | N/A |
|  | 24b | Indicate where the review protocol can be accessed, or state that a protocol was not prepared. | 6 |
|  | 24c | Describe and explain any amendments to information provided at registration or in the protocol. | N/A |
| Support | 25 | Describe sources of financial or non-financial support for the review, and the role of the funders or sponsors in the review. | N/A |
| Competing interests | 26 | Declare any competing interests of review authors. | N/A |
| Availability of data, code and other materials | 27 | Report which of the following are publicly available and where they can be found: template data collection forms; data extracted from included studies; data used for all analyses; analytic code; any other materials used in the review. | Table 1 |

*From:*  Page MJ, McKenzie JE, Bossuyt PM, Boutron I, Hoffmann TC, Mulrow CD, et al. The PRISMA 2020 statement: an updated guideline for reporting systematic reviews. BMJ 2021;372:n71. doi: 10.1136/bmj.n71

For more information, visit: <http://www.prisma-statement.org/>

| **Section and Topic** | **Item #** | **Checklist item** | **Reported (Yes/No)** |
| --- | --- | --- | --- |
| **TITLE** | | |  |
| Title | 1 | Identify the report as a systematic review and/or meta-analysis. | Yes |
| **BACKGROUND** | | |  |
| Objectives | 2 | Provide an explicit statement of the main objective(s) or question(s) the review addresses. | Yes |
| **METHODS** | | |  |
| Eligibility criteria | 3 | Specify the inclusion and exclusion criteria for the review. | Yes |
| Information sources | 4 | Specify the information sources (e.g. databases, registers) used to identify studies and the date when each was last searched. | Yes |
| Risk of bias | 5 | Specify the methods used to assess risk of bias in the included studies. | Yes |
| Synthesis of results | 6 | Specify the methods used to present and synthesise results. | Yes |
| **RESULTS** | | |  |
| Included studies | 7 | Give the total number of included studies and participants and summarise relevant characteristics of studies. | Yes |
| Synthesis of results | 8 | Present results for main outcomes, preferably indicating the number of included studies and participants for each. If meta-analysis was done, report the summary estimate and confidence/credible interval. If comparing groups, indicate the direction of the effect (i.e. which group is favoured). | Yes |
| **DISCUSSION** | | |  |
| Limitations of evidence | 9 | Provide a brief summary of the limitations of the evidence included in the review (e.g. study risk of bias, inconsistency and imprecision). | Yes |
| Interpretation | 10 | Provide a general interpretation of the results and important implications. | Yes |
| **OTHER** | | |  |
| Funding | 11 | Specify the primary source of funding for the review. | No |
| Registration | 12 | Provide the register name and registration number. | No |

*From:*  Page MJ, McKenzie JE, Bossuyt PM, Boutron I, Hoffmann TC, Mulrow CD, et al. The PRISMA 2020 statement: an updated guideline for reporting systematic reviews. BMJ 2021;372:n71. doi: 10.1136/bmj.n71

For more information, visit: <http://www.prisma-statement.org/>

# **Table 2.** Literature search strategy

| Set# | Search term |
| --- | --- |
| S1 | tio,ab(Long COVID*) |
| S2 | EMB.EXACT("long-haul COVID") |
| S3 | MESH.EXACT("Post-Acute COVID-19 Syndromes") |
| S4 | EMB.EXACT.EXPLODE("Long COVID ") OR EMB.EXACT("long-haul COVID") |
| S5 | MESH.EXACT.EXPLODE("Long COVID") OR MESH.EXACT.EXPLODE("Post-Acute COVID-19 Syndromes") OR MESH.EXACT("3 years") OR MESH.EXACT("Three-year") |
| S6 | ti,ab(("Long COVID*" or long-haul COVID* or " Post-Acute COVID-19 Syndromes*" or " 3 years *" or " Three-year*")) |
| S7 | EMB.EXACT.EXPLODE("CORONAVIRIDAE INFECTION") OR EMB.EXACT.EXPLODE("CORONAVIRUS INFECTIONS") |
| S8 | tio,ab(“COVID-19” or “SARS-CoV-2” or “2019-nCoV” or “n-CoV” and “coronavirus”) |
| S9 | S5 OR S4 OR S3 OR S2 OR S1 |
| S10 | S9 OR S8 OR S7 OR S6 |
| S11 | S10 OR S9 |
| S12 | S11 AND S10 AND S9 |

# **Table 3.** A list of the excluded studies and reasons for their exclusion

|  | **Studies** | **Exclusion Reason** |
| --- | --- | --- |
| 1 | Alrasheedi A. The prevalence of covid-19 in the countries of the gulf cooperation council: an examination after three years. Georgian Medical News. 2023 Feb 1(335):6-12. | Unsuitable outcomes |
| 2 | Jansen EB, Ostadgavahi AT, Hewins B, Buchanan R, Thivierge BM, Sganzerla Martinez G, Goncin U, Francis ME, Swan CL, Scruten E, Bell J. PASC (Post Acute Sequelae of COVID-19) is associated with decreased neutralizing antibody titers in both biological sexes and increased ANG-2 and GM-CSF in females. Scientific Reports. 2024 Apr 29;14(1):9854. | Follow-up < 3 years |
| 3 | Lam IC, Wong CK, Zhang R, Chui CS, Lai FT, Li X, Chan EW, Luo H, Zhang Q, Man KK, Cheung BM. Long-term post-acute sequelae of COVID-19 infection: a retrospective, multi-database cohort study in Hong Kong and the UK. EClinicalMedicine. 2023 Jun 1;60. | Follow-up < 3 years |
| 4 | Lythgoe KA, Golubchik T, Hall M, House T, Cahuantzi R, MacIntyre-Cockett G, Fryer H, Thomson L, Nurtay A, Ghafani M, Buck D. Lineage replacement and evolution captured by 3 years of the United Kingdom Coronavirus (COVID-19) Infection Survey. Proceedings of the Royal Society B. 2023 Oct 18;290(2009):20231284. | Unsuitable outcomes |
| 5 | Mahadeva U, Vigliar E. COVID‐19 and cytopathology: The many faces of the pandemic's impact 3 years on. Cytopathology. 2023 Sep;34(5):404-5. | Editorial |
| 6 | Otani K, Fukushima H, Matsuishi K. COVID-19 delirium and encephalopathy: Pathophysiology assumed in the first 3 years of the ongoing pandemic. Brain Disorders. 2023 Jun 1;10:100074. | Review |
| 7 | Pellegrino CJ, Ye NY, Chen IK, Abdelsayed GA. Early and late implications of COVID-19 on male reproductive health: 3 years of data. Sexual Medicine Reviews. 2023 Jul;11(3):224-30. | Unsuitable outcomes |
| 8 | Peluso MJ, Ryder D, Flavell R, Wang Y, Levi J, LaFranchi BH, Deveau TM, Buck AM, Munter SE, Asare KA, Aslam M. Multimodal molecular imaging reveals tissue-based T cell activation and viral RNA persistence for up to 2 years following COVID-19. Medrxiv. 2023 Jan 1. | Follow-up < 3 years |
| 9 | Rao S, Gross RS, Mohandas S, Stein CR, Case A, Dreyer B, Pajor NM, Bunnell HT, Warburton D, Berg E, Overdevest JB. Postacute sequelae of SARS-CoV-2 in children. Pediatrics. 2024 Mar 1;153(3):e2023062570. | Review |
| 10 | Taquet M, Skorniewska Z, De Deyn T, Hampshire A, Trender WR, Hellyer PJ, Chalmers JD, Ho LP, Horsley A, Marks M, Poinasamy K. Cognitive and psychiatric symptom trajectories 2–3 years after hospital admission for COVID-19: a longitudinal, prospective cohort study in the UK. The Lancet Psychiatry. 2024 Jul 31. | Follow-up < 3 years |

# **Table 4.** Quality assessment and publication bias evaluation of included study using the Newcastle-Ottawa Scale (NOS).

| **Cohort study** | **Selection (4)** |  |  |  | **Comparability (2)** | | **Outcome (3)** | |  | **Total score** |
| --- | --- | --- | --- | --- | --- | --- | --- | --- | --- | --- |
| **Author** | **Representativeness of exposed cohort** | **Selection** | **Ascertainment of exposure** | **Demonstration that outcome of interest was not present at the start of the study** | **Study control for confounding factors** | **Additional factors; controlled for ≥ 2 variables including comorbidities** | **Assessment of outcome** | **Was follow-up long enough for outcomes to occur** | **Adequacy of follow-up of cohorts** |  |
| Boscolo-Rizzo et al. 2023 | √ | - | - | √ | √ | √ | √ | √ | √ | 7 |
| Bota et al. 2024 | √ | - | - | √ | √ | √ | √ | √ | √ | 7 |
| Cai et al. 2024 | √ | √ | √ | √ | √ | √ | √ | √ | √ | 9 |
| Dai et al. 2024 | √ | - | √ | √ | √ | √ | √ | √ | √ | 8 |
| Eligulashvili et al. 2024 | √ | - | √ | √ | √ | √ | √ | √ | √ | 8 |
| Filev et al. 2024 | √ | - | - | √ | √ | √ | √ | √ | √ | 7 |
| Han et al. 2024 | √ | - | √ | √ | √ | √ | √ | √ | √ | 8 |
| Morioka et al. 2024 | - | - | √ | √ | √ | √ | √ | √ | - | 6 |
| Wu et al. 2024 | √ | √ | √ | √ | √ | √ | √ | √ | √ | 9 |
| Yang et al. 2024 | √ | - | √ | √ | √ | √ | √ | √ | √ | 8 |
| Zhang et al. 2023 | √ | √ | √ | √ | √ | √ | √ | √ | √ | 9 |

# **Table 5.** Prevalence of different outcomes 3 years after SARS-CoV-2 infection.

| **Organ** | **Outcome** | **Prevalence** | **Comparaison** | **Study** |
| --- | --- | --- | --- | --- |
| **Neurologic** | Alzheimer’s disease | (IRR: 1.76 (95% CI: 1.21-2.57) | Control group without infection | Cai et al. 2024 |
|  | Mobility problem | (IRR: 1.32 (95% CI: 1. 1-1.59) | Control group without infection | Cai et al. 2024 |
|  | Mobility problem | (33/752 [4%]) | NA | Zhang et al. 2023 |
|  | Dizziness | (77/1358 [6%]), | NA | Zhang et al. 2023 |
|  | Dizziness | (IRR: 1.43 (95% CI: 1.26-1.61) | Control group without infection | Cai et al. 2024 |
|  | Dysautonomia | (IRR: 1.46 (95% CI: 1.17-1.8) | Control group without infection | Cai et al. 2024 |
|  | Epilepsy/seizure | (IRR: 2.06 (95% CI: 1.62-2.61) | Control group without infection | Cai et al. 2024 |
|  | Headache | (IRR: 1.17 (95% CI: 0.9-1.52) | Control group without infection | Cai et al. 2024 |
|  | Headache | (61/1358 [4%]). | NA | Zhang et al. 2023 |
|  | Ischemic stroke | (28/1196 [2.34%]) | NA | Eligulashvili et al. 2024 |
|  | Ischemic stroke | (IRR: 1.57 (95% CI: 1.3-1.91) | Control group without infection | Cai et al. 2024 |
|  | Loss of smell | (IRR: 5.56 (95% CI: 3.61-8.56) | Control group without infection | Cai et al. 2024 |
|  | Loss of smell | (86/1358 [6%]) | NA | Zhang et al. 2023 |
|  | Loss of smell | (12/88 [13.6%]) | NA | Boscolo-Rizzo et al. 2023 |
|  | Loss of taste | (10/88 [11.4%]) | NA | Boscolo-Rizzo et al. 2023 |
|  | Loss of taste | (51/1358 [4%]) | NA | Zhang et al. 2023 |
|  | Memory problems | (IRR: 1.76 (95% CI: 1.21-2.57) | Control group without infection | Cai et al. 2024 |
|  | Sleep difficulties | (370/1358 [27%]) | NA | Zhang et al. 2023 |
|  | Parkinson's-like disease | (IRR: 1.55 (95% CI: 1.08-2.21) | Control group without infection | Cai et al. 2024 |
|  | Peripheral neuropathy | (IRR: 1.16 (95% CI: 1.02-1.33) | Control group without infection | Cai et al. 2024 |
|  | Vision abnormalities | (IRR: 1.15 (95% CI: 1.00-1.31) | Control group without infection | Cai et al. 2024 |
| **Mental** | Acute stress | (IRR: 2.17 (95% CI: 1.45-3.26) | Control group without infection | Cai et al. 2024 |
|  | Adjustment disorder | (IRR: 1.14 (95% CI: 0.99-1.32) | Control group without infection | Cai et al. 2024 |
|  | Alcohol use disorder | (IRR: 1.31 (95% CI: 1.11-1.55) | Control group without infection | Cai et al. 2024 |
|  | Anxiety | (IRR: 1.5 (95% CI: 1.18-1.9) | Control group without infection | Cai et al. 2024 |
|  | Anxiety | (156/752 [21%]) | NA | Zhang et al. 2023 |
|  | Depression | (IRR: 1.27 (95% CI: 1.1-1.47) | Control group without infection | Cai et al. 2024 |
|  | Depression | (278/752 [37%]) | NA | Zhang et al. 2023 |
|  | Illicit drug use disorder | (IRR: 1.39 (95% CI: 1.16-1.67) | Control group without infection | Cai et al. 2024 |
|  | Nicotine use disorder | (IRR: 1.27 (95% CI: 1.11-1.46) | Control group without infection | Cai et al. 2024 |
|  | Opioid use disorder | (IRR: 1.83 (95% CI: 1.39-2.43) | Control group without infection | Cai et al. 2024 |
|  | Post-traumatic stress disorder symptom | (IRR: 1.2 (95% CI: 1.05-1.37) | Control group without infection | Cai et al. 2024 |
|  | Post-traumatic stress disorder symptom | (120/16 [52%]) | NA | Zhang et al. 2023 |
|  | Panic disorder | (IRR: 1.11 (95% CI: 0.79-1.54) | Control group without infection | Cai et al. 2024 |
|  | Sedatives/ hypnotics use disorder | (IRR: 2.42 (95% CI: 1.45-4.02) | Control group without infection | Cai et al. 2024 |
|  | Sleep disorder | (IRR: 1.3 (95% CI: 1.19-1.42) | Control group without infection | Cai et al. 2024 |
|  | Suicidal ideation | (IRR: 2.02 (95% CI: 1.69-2.42) | Control group without infection | Cai et al. 2024 |
| **Fatigue** | Fatigue | (IRR: 1.43 (95% CI: 1.05-1.94) | Control group without infection | Cai et al. 2024 |
|  | Fatigue | (249/1358 [18%]) | NA | Zhang et al. 2023 |
| **Pulmonary** | Cough | (IRR: 1.88 (95% CI: 1.7-2.07) | Control group without infection | Cai et al. 2024 |
|  | Dyspnea | (IRR: 1.67 (95% CI: 1.53-1.83) | Control group without infection | Cai et al. 2024 |
|  | Hypoxemia | (IRR: 1.93 (95% CI: 1.57-2.38) | Control group without infection | Cai et al. 2024 |
|  | Interstitial lung disease | (IRR: 2.62 (95% CI: 2.08-3.31) | Control group without infection | Cai et al. 2024 |
| **Cardiovascular** | Acute coronary disease | (IRR: 1.58 (95% CI: 1.4-1.79) | Control group without infection | Cai et al. 2024 |
|  | Angina | (IRR: 1.93 (95% CI: 1.58-2.35) | Control group without infection | Cai et al. 2024 |
|  | Atrial fibrillation | (IRR: 1.21 (95% CI: 1.02-1.45) | Control group without infection | Cai et al. 2024 |
|  | Atrial flutter | (IRR: 1.3 (95% CI: 0.83-2.05) | Control group without infection | Cai et al. 2024 |
|  | Bradycardia | (IRR: 1.71 (95% CI: 1.47-2.00) | Control group without infection | Cai et al. 2024 |
|  | Cardiac arrest | (IRR: 1.71 (95% CI: 0.9-3.26) | Control group without infection | Cai et al. 2024 |
|  | Heart failure | (IRR: 1.63 (95% CI: 1.43-1.85) | Control group without infection | Cai et al. 2024 |
|  | Ischemic cardiomyopathy | (IRR: 1.39 (95% CI: 1.01-1.92) | Control group without infection | Cai et al. 2024 |
|  | major adverse cardiac events | (198/1196 [16.51%]) | NA | Eligulashvili et al. 2024 |
|  | Myocardial infraction | (IRR: 1.58 (95% CI: 1.4-1.78) | Control group without infection | Cai et al. 2024 |
|  | Myocardities | (IRR: 2.07 (95% CI:0.39-10.97) | Control group without infection | Cai et al. 2024 |
|  | Nonischemic cardiomyopathy | (IRR: 1.65 (95% CI: 1.34-2.04) | Control group without infection | Cai et al. 2024 |
|  | Palpitation | (123/1358 [9%]). | NA | Zhang et al. 2023 |
|  | Pericarditis | (IRR: 1.18 (95% CI: 0.53-2.6) | Control group without infection | Cai et al. 2024 |
|  | Tachycardia | (IRR: 1.82 (95% CI: 1.54-2.15) | Control group without infection | Cai et al. 2024 |
|  | Ventricular arrhythmia | (IRR: 1.92 (95% CI: 1.52-2.41) | Control group without infection | Cai et al. 2024 |
| **Musculoskeletal** | Joint pain | (IRR: 1.35 (95% CI: 1.25-1.46) | Control group without infection | Cai et al. 2024 |
|  | Myalgia | (IRR: 1.34 (95% CI: 1.12-1.6) | Control group without infection | Cai et al. 2024 |
|  | Myalgia | (90/1358 [7%]) | NA | Zhang et al. 2023 |
|  | Osteoarthritis | (IRR: 1.13 (95% CI: 1.02-1.25) | Control group without infection | Cai et al. 2024 |
| **Kidney** | acute kidney injury | (IRR: 2.13 (95% CI: 1.89-2.41) | Control group without infection | Cai et al. 2024 |
|  | estimated glomerular filtration rate decline >30% | (IRR: 1.27 (95% CI: 1.16-1.39) | Control group without infection | Cai et al. 2024 |
| **Gastrointestinal** | Abdominal pain | (IRR: 1.92 (95% CI: 1.75-2.11) | Control group without infection | Cai et al. 2024 |
|  | Acute gastritis | (IRR: 1.38 (95% CI: 1.09-1.74) | Control group without infection | Cai et al. 2024 |
|  | Acute pancreatitis | (IRR: 2.3 (95% CI: 1.61-3.27) | Control group without infection | Cai et al. 2024 |
|  | Constipation | (IRR: 1.4 (95% CI: 1.25-1.57) | Control group without infection | Cai et al. 2024 |
|  | Diarrhea | (IRR: 2.03 (95% CI: 1.82-2.28) | Control group without infection | Cai et al. 2024 |
|  | Gastroesophageal reflux disease | (IRR: 1.26 (95% CI: 1.13-1.39) | Control group without infection | Cai et al. 2024 |
|  | Irritable bowel syndrome | (IRR: 1.43 (95% CI: 1.27-1.62) | Control group without infection | Cai et al. 2024 |
|  | Liver abnormalities | (IRR: 1.57 (95% CI: 1.37-1.8) | Control group without infection | Cai et al. 2024 |
|  | Peptic ulcer disease | (IRR: 1.68 (95% CI: 1.25-2.26) | Control group without infection | Cai et al. 2024 |
|  | Vomiting | (IRR: 2.12 (95% CI: 1.65-2.74) | Control group without infection | Cai et al. 2024 |
| **Metabolic** | Diabetes | (IRR: 1.27 (95% CI: 0.91-1.76) | Control group without infection | Cai et al. 2024 |

IRR, incidence rate ratios; 95% CI, 95% confidence intervals.

# **Figure 1.** Full summary plot for the Newcastle-Ottawa Scale.


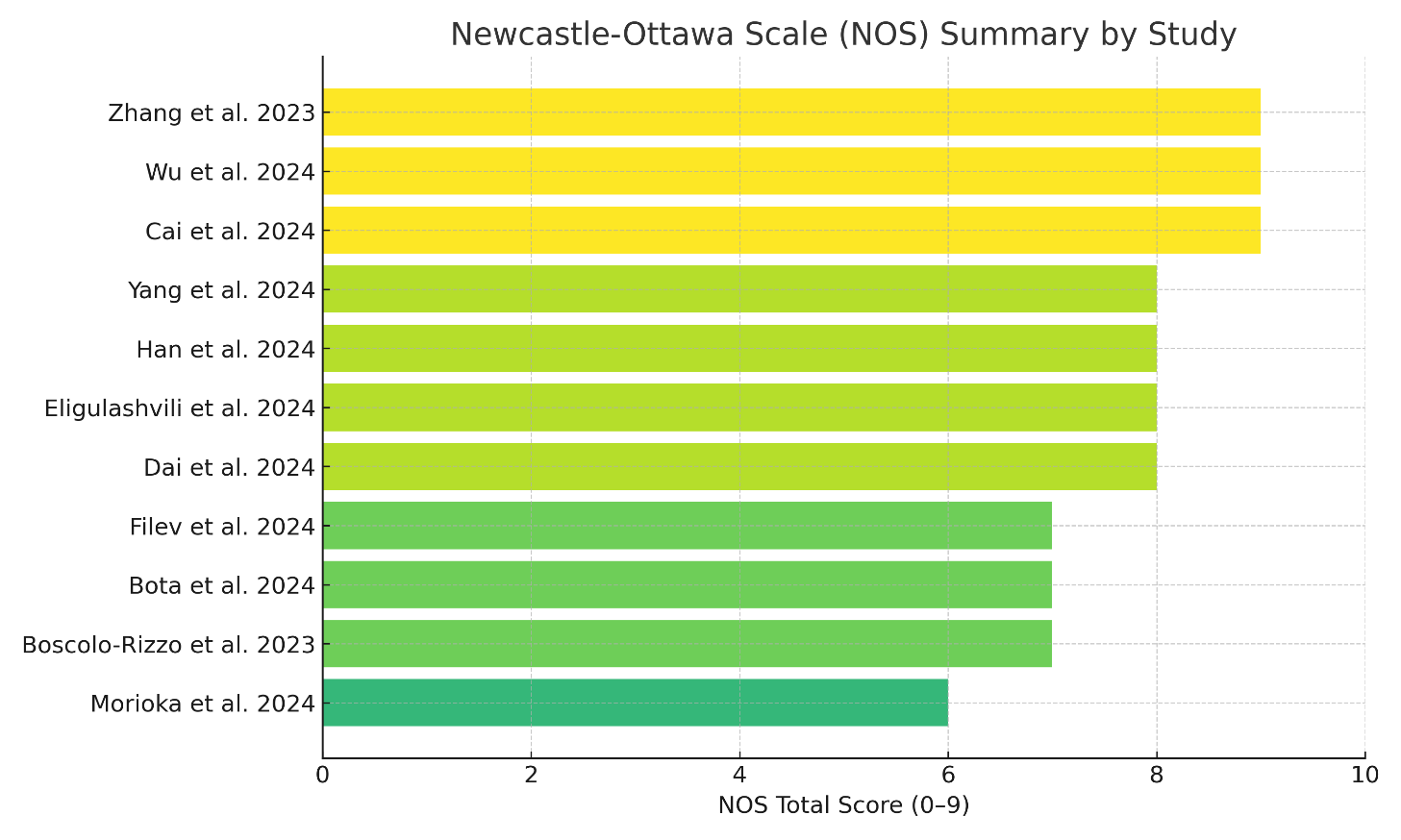

Supplement: Supplementary file 1 — Supplementary materials. [file JMV-97-e70429-s001.docx]
